# A single sequence context cannot satisfy all non-AUG initiator codons in yeast^†^

**DOI:** 10.1186/1471-2180-10-188

**Published:** 2010-07-09

**Authors:** Chia-Pei Chang, Shun-Jia Chen, Chen-Huan Lin, Tzu-Ling Wang, Chien-Chia Wang

**Affiliations:** 1 Department of Life Science, National Central University, 300 Jung-da Rd., Jung-li 32001, Taiwan; 2 Science Education Center, National Taiwan Normal University, 88 Ting-Chou Rd., Sec. 4, Taipei 11677, Taiwan

## Abstract

**Background:**

Previous studies in *Saccharomyces cerevisiae *showed that *ALA1 *(encoding alanyl-tRNA synthetase) and *GRS1 *(encoding glycyl-tRNA synthetase) respectively use ACG and TTG as their alternative translation initiator codons. To explore if any other non-ATG triplets can act as initiator codons in yeast, *ALA1 *was used as a reporter for screening.

**Results:**

We show herein that except for AAG and AGG, all triplets that differ from ATG by a single nucleotide were able to serve as initiator codons in *ALA1*. Among these initiator codons, TTG, CTG, ACG, and ATT had ~50% initiating activities relative to that of ATG, while GTG, ATA, and ATC had ~20% initiating activities relative to that of ATG. Unexpectedly, these non-AUG initiator codons exhibited different preferences toward various sequence contexts. In particular, GTG was one of the most efficient non-ATG initiator codons, while ATA was essentially inactive in the context of *GRS1*.

**Conclusion:**

This finding indicates that a sequence context that is favorable for a given non-ATG initiator codon might not be as favorable for another.

## Background

Aminoacyl-tRNA synthetases are a group of translation enzymes, each of which catalyzes the attachment of a specific amino acid to its cognate tRNAs. The resultant aminoacyl-tRNAs are then delivered by elongation factor (EF)-1 to ribosomes for protein translation. Typically there are 20 different aminoacyl-tRNA synthetases in prokaryotes, one for each amino acid [1-4]. In eukaryotes, protein synthesis occurs in the cytoplasm as well as in organelles, such as mitochondria and chloroplasts [5]. Thus, eukaryotes, such as yeast, need two distinct sets of enzymes for each aminoacylation activity, one localized in the cytoplasm and the other in mitochondria. Each set of enzymes aminoacylates isoaccepting tRNAs within its respective cell compartment. In most cases, cytoplasmic and mitochondrial synthetase activities are encoded by two distinct nuclear genes. However, two *Saccharomyces cerevisiae *genes, *HTS1 *(the gene encoding histidyl-tRNA synthetase) [6] and *VAS1 *(the gene encoding valyl-tRNA synthetase (ValRS)) [7], specify both the mitochondrial and cytosolic forms through alternative translation initiation from two in-frame AUG codons.

A previous study on *CYC1 *of *S. cerevisiae *suggested that AUG is the only codon recognized as a translational initiator, and that the AUG codon nearest the 5' end of the mRNA serves as the start site for translation [8]. If the first AUG codon is mutated, then initiation can begin at the next available AUG from the 5' end of mRNA. The same rules apply to all eukaryotes. However, many examples of non-AUG initiation were reported in higher eukaryotes, where cellular and viral mRNAs can initiate from codons that differ from AUG by one nucleotide [9]. The relatively weak base-pairing between a non-AUG initiator codon and the anticodon of an initiator tRNA appears to be compensated for by interactions with nearby nucleotides, in particular a purine (A or G) at position -3 and a "G" at position +4 [10,11]. A recent study suggested that components of the 48 S translation initiation complex, in particular eIF2 and 18 S ribosomal (r)RNA, might be involved in specific recognition of the -3 and +4 nucleotides [11]. In addition to the sequence context, a stable hairpin structure located 12~15 nucleotides downstream of the initiator can also facilitate recognition of a poor initiator by the 40 S ribosomal subunit [12].

While the sequence context can also modulate the efficiency of AUG initiation in yeast, the magnitude of this effect appears relatively insignificant [13-15]. Perhaps for that reason, yeast cannot efficiently use non-AUG codons as translation start sites [16,17]. Nonetheless, three yeast genes, *GRS1 *(one of the two glycyl-tRNA synthetase (GlyRS) genes in *S. cerevisiae*) [18], *ALA1 *(the only alanyl-tRNA synthetase (AlaRS) gene in *S. cerevisiae*) [19], and *CARP2A *(the gene coding for the acidic ribosomal protein, P2A, in *Candida albicans*) [20], were recently shown to use naturally occurring non-AUG triplets as translation initiators. Moreover, the translational efficiency of non-AUG initiation is deeply affected (by up to 32-fold) by nucleotides at the -3 to -1 relative positions, especially -3. AARuug (R denotes A or G; uug denotes a non-AUG initiation codon) appears to represent the most favorable sequence context [21].

A unique feature of the gene expression of *ALA1 *is that the mitochondrial form of AlaRS is initiated from two consecutive in-frame ACG codons, with the first being more robust [19,22]. Redundant ACGs contain stronger initiation activities than does a single ACG [23]. This feature of recurrence of non-AUG initiator codons may in itself represent a novel mechanism to improve the overall efficiency of translation [24]. To investigate if any other non-AUG triplets can act as initiator codons in yeast, a random triplet was introduced into *ALA1 *to replace the native initiation sites and screened. We show herein that except for AAG and AGG, all other non-AUG codons that differ from AUG by a single nucleotide can functionally substitute for the redundant ACG initiator codons of *ALA1*. These non-AUG initiator codons possessed different initiating activities and exhibited different preferences for various sequence contexts. For example, GTG, a less-efficient non-AUG initiator codon in the context of *ALA1*, was one of the strongest non-AUG initiator codons in the context of *GRS1*. On the contrary, ATA, a fairly active non-AUG initiator codon in the context of *ALA1*, was essentially inactive in the context of *GRS1*. Thus, every non-AUG initiator codon may have its own favorite sequence context in yeast.

## Methods

### Construction of various *ALA1 *and *ALA1*-*lexA *fusion constructs

Cloning of the wild-type (WT) *ALA1 *gene in a low-copy-number yeast shuttle vector, pRS315, was previously described [19]. A 5'-end truncated version of *ALA1*, extending from base pairs +54 to +2877 (relative to ATG1) was amplified by a polymerase chain reaction (PCR) and cloned in the XbaI/XhoI sites of pRS315, yielding pCW415. To mutate the repeating ACG initiator codons of *ALA1*, a short *ALA1 *sequence containing base pairs -250 to +54 was amplified by a PCR as an EagI-XbaI fragment and cloned into the appropriate sites of pBluescript II SK (+/-) (Stratagene, La Jolla, CA). Mutations were created by a PCR-based mutagenesis following the protocols provided by Stratagene. The repeating ACG triplets, ACG(-25)/ACG(-24), were first mutated to GGT(-25)/ACC(-24) to eliminate their initiating activities. A random triplet (designated here as "NNN") was then introduced to replace GGT(-25). The resultant *ALA1 *fragment was recovered from the plasmid by digestion with EagI and XbaI, and then ligated into the EagI/XbaI sites of pCW415, yielding a library of *ALA1 *sequences with differences only at codon position -25.

Construction of various *ALA1-lexA *or *GRS1-lexA *fusion constructs for the Western blot analyses was as previously described [24]. Briefly, an initiator mutant of *lexA *was amplified by PCR as an SpeI-XhoI fragment and cloned in the pADH high-copy-number yeast shuttle vector. A wild-type (WT) or mutant *ALA1 *sequence containing base pairs -105 to -24 relative to ATG1 was amplified by PCR as a PstI-SpeI fragment and was cloned in-frame into the 5' end of *lexA*, resulting in various *ALA1-lexA *fusion constructs. Construction of *GRS1-lexA *fusion constructs followed a similar strategy. The expression of these *lexA *fusion constructs was under the control of a constitutive *ADH *promoter [25]. The Western blot analysis was as previously described [24].

### Complementation assays for the cytoplasmic and mitochondrial functions of *ALA1*

The yeast *ALA1 *knockout strain, TRY11 (*MAT*a, *his3Δ200*, *leu2Δ1*, *lys2-801*, *trp1Δ101*, *ura3-52*, and *ala1Δ::TRP1*) was maintained by a plasmid carrying the WT *ALA1 *gene and a *URA3 *marker [26]. Complementation assays for the cytoplasmic function of plasmid-borne *ALA1 *and its derivatives were carried out by introducing a test plasmid (with a *LEU2 *marker) into TRY11 and determining the ability of transformants to grow in the presence of 5-fluoroorotic acid (5-FOA). Cultures were incubated at 30°C for 3~5 days or until colonies appeared. The transformants evicted the maintenance plasmid that carries the *URA3 *marker in the presence of 5-FOA. Thus, only an enzyme with cytoplasmic AlaRS activity encoded by the test plasmid could rescue the growth defect.

Following 5-FOA selection, a single colony of transformants was selected and grown to the stationary phase in synthetic medium lacking leucine. Starting from a cell density of 1.0 *A*_600_, cultures were 5-fold serially diluted, and 5-μl aliquots of each dilution were spotted onto the designated YPG plates. The plates were incubated at 30°C for 3~5 days. Photos were taken of the complementation assays on day 3 following incubation. Because yeast cells cannot survive on glycerol without functional mitochondria, the transformants did not grow on YPG plates unless a functional mitochondrial AlaRS was generated by the test plasmid. Assays of the cytoplasmic and mitochondrial GlyRS activities followed a similar protocol [21].

### Reverse-transcription (RT)-PCR

To determine the relative levels of specific *ALA1-lexA *mRNAs derived from the fusion constructs, a semiquantitative RT-PCR experiment was carried out following the protocols provided by the manufacturer (Invitrogen). Briefly, total RNA was first isolated from the transformants, and aliquots (~1 μg) of RNA were then reverse-transcribed into single-stranded complementary (c)DNA using an oligo-dT primer. After RNase H treatment, the single-stranded cDNA products were amplified by a PCR using a pair of specific primers. The forward and reverse primers contained sequences complementary to nucleotides -90 to -70 of *ALA1 *(5'-TATGAAAGCAGTTTGATTGAA-3') and nucleotides +370 to +390 of *lexA *(5'-CAAGTCACCATCCATAATGCC-3'), respectively. Two different cycle numbers of PCR amplification were carried out for each cDNA preparation as indicated in the figure. As a control, the relative levels of actin-specific mRNAs in each preparation were also determined using a set of primers complementary to nucleotides +537 to +560 (5'-ACCAACTGGGACGATATGGAAAAG-3') and nucleotides +696 to +719 (5'-TTGGATGGAAACGTAGAAGGCTGG-3') of actin, respectively. Determination of the relative levels of specific *GRS1-lexA *mRNAs derived from the fusion constructs followed a similar protocol [21].

### β-Galactosidase (gal) assay

Yeast cells were pelleted by centrifugation at 12,000 ×*g *for 30 s and resuspended in 100 μl of breaking buffer (100 mM Tris-HCl (pH 8.0), 1 mM DTT, 10% glycerol, and 2 mM PMSF) and 100 μl of beads. Cells were then lysed at 4°C using a bead beater, followed by centrifugation at 12,000 ×*g *for 2 min. Aliquots of the supernatants (25~250 μg) were diluted to 0.8 ml with Z buffer (60 mM Na_2_HPO_4_, 40 mM NaH_2_PO_4_, 10 mM KCl, 1 mM MgSO_4_, and 50 mM 2-ME). β-Gal activity assays were initiated (at 37°C) by adding 0.2 ml of o-nitrophenyl β-D-galactoside (4 mg/ml). The reaction mixtures were incubated with constant shaking at 37°C for 20 min and then terminated by the addition of 0.4 ml of 1 M Na_2_CO_3_. The reaction mixtures were centrifuged at 12,000 ×*g *for 2 min, and the absorbance (*A*_420_) of the supernatants was determined. Relative β-gal activities were calculated from *A*_420 _readings normalized to protein concentrations.

## Results

### Screening for functional non-AUG initiator codons using *ALA1 *as a reporter

**Figure 1:**
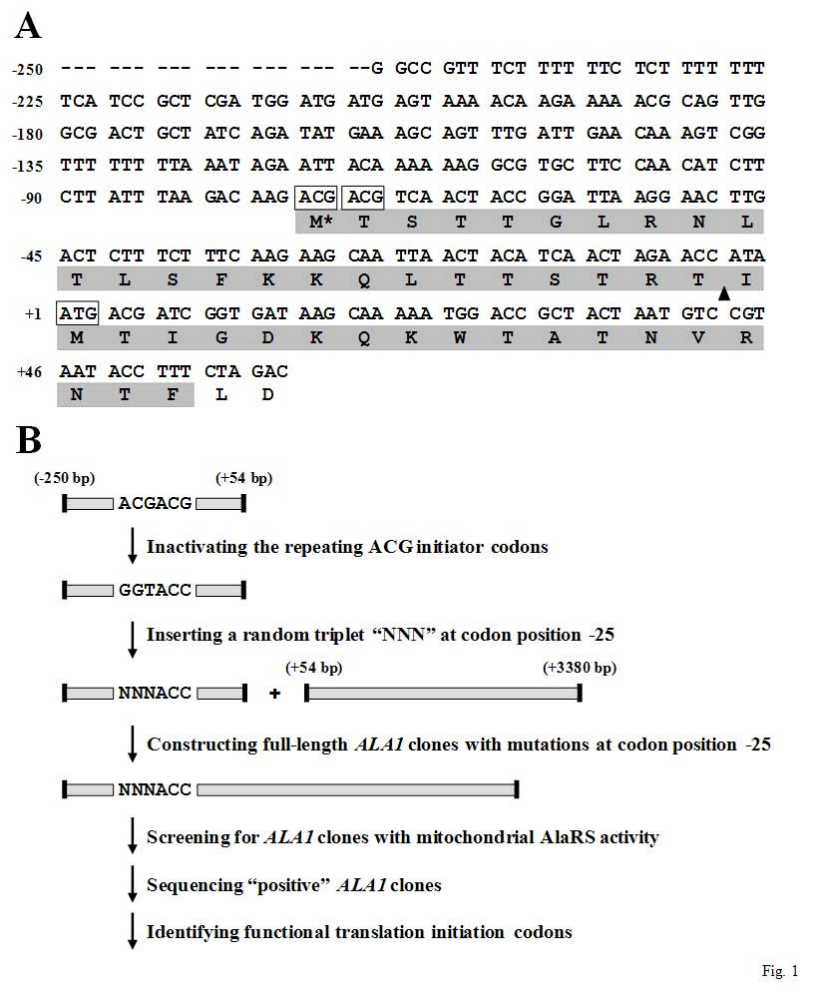
**Screening for all feasible non-AUG initiation codons**. (A) Nucleotide sequences -250 to +60 relative to ATG1 of *ALA1*. For clarity, the translation initiation codons ACG(-25)/ACG(-24) and ATG1 are boxed, and the mitochondrial targeting signal is shaded. The amino acid residue encoded by ACG(-25) is labeled M*. The cleavage site for the mitochondrial matrix-processing peptidase is marked under the sequence by a black triangle (▲). (B) Screening for feasible non-AUG initiator codons.

Our previous study [19] showed that two successive in-frame ACG triplets 23 codons upstream of the ATG1 initiator codon, *i.e*., ACG(-25) and ACG(-24), serve as translational start sites of the mitochondrial form of AlaRS (Figure 1A). Because examples of naturally occurring non-AUG initiation are still rare in lower eukaryotes, we wondered whether any other non-AUG triplet could function as a translation start site in yeast. To shed new light on this query, an in vivo screening protocol using *ALA1 *as a reporter gene was accordingly designed (see Figure 1B). Briefly, a short *ALA1 *sequence containing base pairs -250 to +54 relative to ATG1 was amplified by PCR as an EagI/XbaI fragment and cloned in the corresponding sites of pBluescript II SK (+/-). The repeating ACG initiator codons in this short fragment were first inactivated by mutation to codons unsuitable for initiation, *i.e*., GGT(-25)/ACC(-24). A random triplet (designated here as "NNN") was subsequently introduced to replace GGT(-25), resulting in NNN(-25)/ACC(-24). The *ALA1 *fragment containing a random triplet at codon position -25 was recovered from the plasmid by EagI/XbaI digestion and fused in-frame to a 5' truncated *ALA1 *gene (extending from base pairs +54 to +2877), yielding a library of *ALA1 *constructs with differences only at codon position -25.

This library of *ALA1 *constructs was transformed into an *ala1^- ^*yeast strain, TRY11, and the transformants were streaked on selection medium lacking uracil and leucine. Colonies that grew on the selection medium were picked (1000 colonies were picked) and individually streaked on plates containing 5-FOA. Since the AUG1 initiator codon of the cytoplasmic form of AlaRS remained unchanged, all transformants that contained a full-length *ALA1 *construct were expected to express the cytoplasmic enzyme and survive 5-FOA selection. As it turned out, 592 of 1000 transformants were able to grow on FOA plates, suggesting that ~60% of the *ALA1 *constructs were full length. To investigate which codon at position -25 has the potential to serve as a translation start site of the mitochondrial form, the growth phenotypes of the transformants that survived 5-FOA selection were further tested on YPG plates. On day 3 following streaking, 104 of 592 transformants had grown on the plates. Plasmid DNAs were subsequently recovered from the "positive" clones and sequenced (Figure 1).

### Identification of non-AUG initiator codons

**Figure 2:**
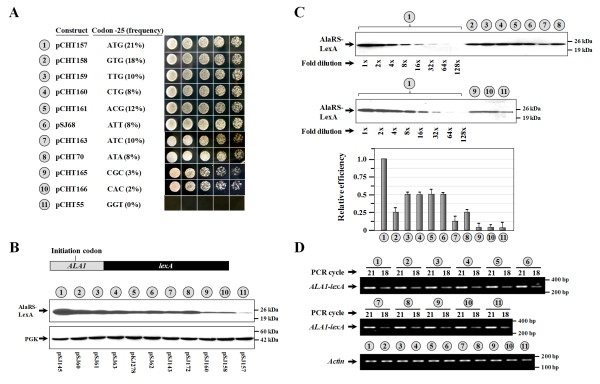
**Comparing the efficiencies of various non-AUG initiator codons in *ALA1***. (A) Complementation assays for mitochondrial AlaRS activity. The *ala1^- ^*strain was transformed with various *ALA1 *constructs, and the growth phenotypes of the transformants were tested. Complementation of the mitochondrial defect of the *ala1^- ^*strain was shown by its ability to lose the maintenance plasmid following FOA selection and grow on a YPG plate. The frequency of each non-AUG initiator codon that appeared in the screening is indicated in the parenthesis behind the codon. (B) Assay of initiating activity by Western blots. *Upper panel*, AlaRS-LexA fusion; *lower panel*, PGK (as loading controls). (C) Assay of the relative initiating activity by Western blots. Protein extracts prepared from the construct with an ATG initiator codon were 2-fold serially diluted and compared to those from constructs with non-ATG initiator codons. The quantitative data for the relative expression levels of these constructs are shown as a separate diagram at the *bottom*. (D) RT-PCR. Relative amounts of specific *ALA1-lexA *mRNAs generated from each construct were determined by RT-PCR. As a control, relative amounts of actin mRNAs were also determined. The *ALA1 *sequences used in *ALA1-lexA *constructs 1~11 in (B) were respectively transferred from constructs 1~11 shown in (A). In (C) and (D) the numbers 1~11 (circled) denote constructs shown in (B).

As summarized in Figure 2A, 10 different triplets were identified at codon position -25 among these positive clones, including ATG, GTG, TTG, CTG, ACG, ATT, ATC, ATA, CGC, and CAC (Figure 2A, numbers 1~10). It was not surprising to find that GTG, TTG, CTG, ACG, ATT, ATC, and ATA were among initiator candidates, due to their close resemblance to ATG, as each of these triplets differed from ATG by just a single nucleotide. However, it was surprising to find that CGC and CAC were also among the preliminary pool of initiator candidates. The nucleotide sequences of these two triplets are completely divergent from ATG and have never previously been shown to be able to serve as initiator codons in a cap-dependent translational process in any organism. GGT served as a negative control in the assay (Figure 2A, number 11). It should be noted that while AAG and AGG also differed from ATG by a single nucleotide, these two triplets could not serve as initiator codons under similar conditions (data not shown). Perhaps this was because the middle bases in the two initiator codons and in the anticodon are all purines, and a purine pair cannot fit into an A-form helix.

To compare the initiation activities of these non-AUG initiator codons, we chose *lexA *as a reporter. An *ALA1 *fragment containing base pairs -105 to -24 was PCR-amplified from each of these positive clones and fused in-frame to the 5' end of an initiator mutant of *lexA*, yielding various *ALA1-lexA *fusion constructs. These fusion constructs were expressed under the control of a constitutive *ADH *promoter. Since the initiator candidates present in the *ALA1 *portion are the only available initiator codons for these fusion constructs, the relative expression levels of the AlaRS-LexA construct are likely to reflect the initiation activities of these initiator candidates. Figure 2B shows that TTG, CTG, ACG, and ATT had the highest initiating activity, at ~50% relative to that of ATG; GTG, ATC, and ATA had medium initiating activities, at ~20% relative to that of ATG; and CGC and CAC had the lowest initiating activities, at ~5% relative to that of ATG (Figure 2B, C, numbers 1~10). In contrast, GGT had almost no detectable initiating activity (Figure 2B, C, number 11). It was interesting to note that while the CGC and CAC mutants expressed ~20-fold less protein than did the ATG mutant, this level of AlaRS was still sufficient to restore the growth phenotype of the *ALA1 *knockout strain on YPG plates (Figure 2A).

To investigate whether these constructs expressed similar levels of mRNA, a semiquantitative RT-PCR experiment was carried out. Figure 2D shows that similar levels of cDNA products were amplified from transformants carrying these constructs, suggesting that these mutations did not affect the stability of the mRNAs derived from these constructs.

### CGC and CAC *per se *could not act as translation initiator codons

As CGC and CAC have no resemblance to AUG, it is hard to imagine that either of these two triplets can stably pair with the anticodon of an initiator tRNA, even with the help of an optimal sequence context, to trigger the necessary conformational changes of the ternary complex (Met-tRNA(i)(Met)-eIF2-GTP) and subsequent GTP hydrolysis. To ascertain that translation of these two *ALA1 *mutants was actually initiated from CGC or CAC, and not from other remedial initiation sites, codons in the leader sequence that have the potential to serve as secondary translation initiation sites and initiate the synthesis of at least part of the mitochondrial targeting sequence were targeted for mutagenesis, and the protein expression and complementation activity of the resultant mutants were then tested. In this regard, TTG(-16) appeared to be a promising candidate on account of its favorable sequence context.

**Figure 3:**
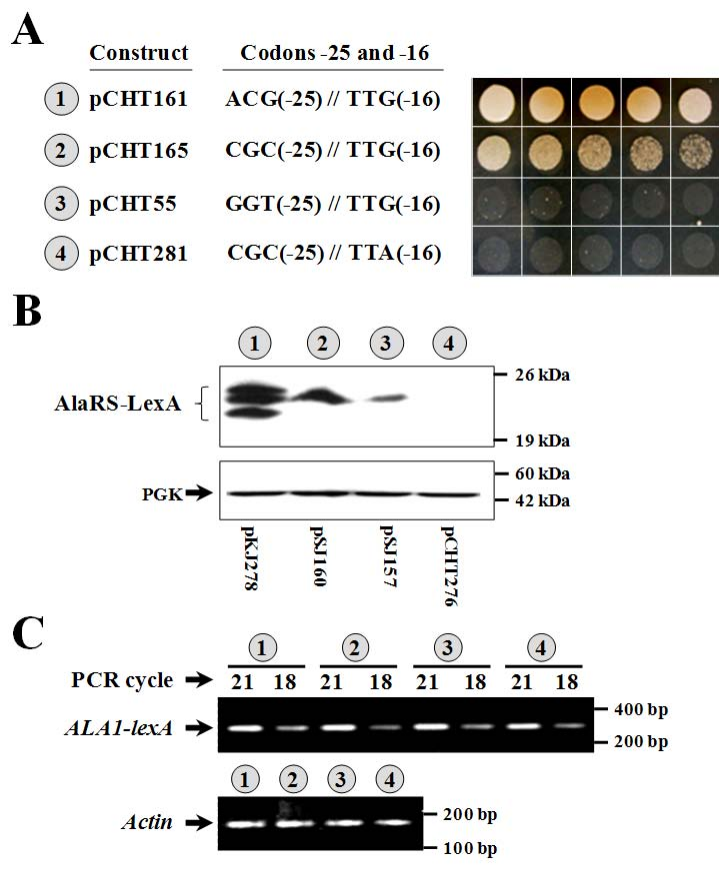
**Rescuing a cryptic translation initiation site in *ALA1***. (A) Complementation assays for mitochondrial AlaRS activity. (B) Assay of initiating activity by Western blots. *Upper panel*, AlaRS-LexA fusion; *lower panel*, PGK (as loading controls). (C) RT-PCR. Relative amounts of specific *ALA1-lexA *mRNAs generated from each construct were determined by RT-PCR. As a control, relative amounts of actin mRNAs were also determined. The *ALA1 *sequences used in the *ALA1-lexA *constructs 1~4 in (B) were respectively transferred from constructs 1~4 shown in (A). In (C) the numbers 1~4 (circled) denote constructs shown in (B).

To distinguish the protein forms initiated from ACG(-25) and UUG(-16), an 18% polyacrylamide gel was used. As shown in Figure 3, mutation of ACG(-25) to CGC had only a minor effect on mitochondrial activity, but drastically reduced protein expression (Figure 3A, B, numbers 1 and 2). The upper and lower protein bands were abolished by the mutation, while the middle band was largely unaffected. This result suggests that both the upper and lower bands were initiated from ACG(-25), and the lower band was derived from cleavage of the upper band possibly by a matrix-processing peptidase. A further mutation that changed TTG(-16) to TTA impaired both the mitochondrial activity and protein expression of the CGC mutant (Figure 3A, B, numbers 2 and 4), suggesting that UUG(-16) served as a remedial initiation site in the CGC mutant and the middle band was initiated from UUG(-16). As the UUG codon possesses stronger initiating activity in the CGC mutant than in the GGU mutant (Figure 3B, numbers 2 and 3), it is possible that CGC(-25) rescued the initiating activity of UUG(-16). Note that the TTG-to-TTA change is a silent mutation and therefore does not affect the stability of the protein form initiated from ACG(-25). A semiquantitative RT-PCR experiment further demonstrated that these mutations at codon position -25 or -16 did not affect the stability of the mRNAs derived from these constructs (Figure 3C).

### Initiation activities of various non-AUG initiator codons in *GRS1*

**Figure 4:**
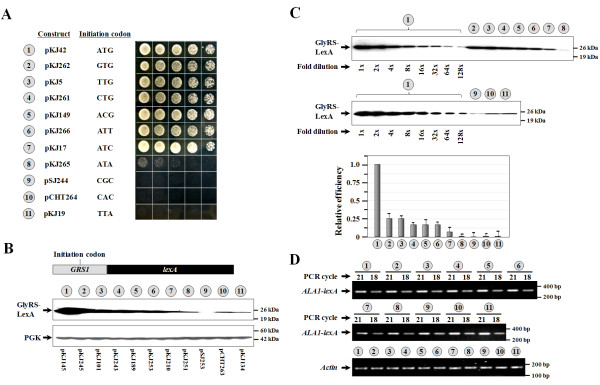
**Comparing the efficiencies of various non-AUG initiator codons in *GRS1***. (A) Complementation assays for mitochondrial GlyRS activity. The *grs1^- ^*strain was transformed with various *GRS1 *constructs, and the growth phenotypes of the transformants were tested. (B) Assay of initiating activities by Western blots. *Upper panel*, GlyRS-LexA fusion; *lower panel*, PGK (as loading controls). (C) Assay of the relative initiating activities by Western blots. Protein extracts prepared from the construct with an ATG initiator codon were 2-fold serially diluted and compared to those from constructs with non-ATG initiator codons. The quantitative data for the relative expression levels of these constructs are shown as a separate diagram at the *bottom*. (D) RT-PCR. Relative amounts of specific *GRS1-lexA *mRNAs generated from each construct were determined by RT-PCR. The *GRS1 *sequences used in the *GRS1-lexA *fusion constructs 1~11 in (B) were respectively transferred from constructs 1~11 shown in (A). In (C) and (D) the numbers 1~11 (circled) denote constructs shown in (B).

We next tested whether the non-AUG initiator codons identified above can also act as initiator codons in *GRS1*. To this end, the native UUG initiator codon of *GRS1 *was substituted by the above-mentioned initiator candidates, and the mitochondrial activities of the resultant mutants were tested. As expected, mutations of TTG(-23) of *GRS1 *to ATG, GTG, CTG, ACG, ATC, or ATT had little effect on mitochondrial activity; transformants carrying any of these mutants grew as well as those carrying a WT *GRS1 *construct on YPG plates (Figure 4A, numbers 1~8). However, a mutation of TTG(-23) to ATA yielded a construct that failed to support the growth of the knockout strain on YPG plates (Figure 4A, number 8). Also, neither CGC nor CAC could act as an initiator codon in *GRS1 *(Figure 4A, numbers 9 and 10). TTA served as a negative control in this assay (Figure 4A, number 11).

To compare the initiating activities of these non-AUG initiator candidates in the context of *GRS1*, a WT or mutant *GRS1 *sequence containing base pairs -88 to -12 relative to ATG1 was fused in-frame to an initiator mutant of *lexA*, and the protein expression levels of these fusion constructs were determined by Western blotting. As shown in Figure 4B and 4C, except for ATA, the often-seen non-AUG initiator candidates possessed 10%~30% initiation activities relative to that of ATG (numbers 1~8). Interestingly, ATA expressed < 2% initiation activity relative to that of ATG (number 8), which provides a rational basis for the negative growth phenotype of the ATA mutant in the functional assay (Figure 4A, number 8). Additionally, it was noted that GTG, a less-efficient non-ATG initiator codon in the context of *ALA1 *(Figure 2C), was one of the most efficient non-ATG initiator codons in the context of *GRS1 *(Figure 4C). As expected, no detectable initiating activity was found for the CGC or CAC mutant under the conditions used (Figure 4B, C, numbers 9 and 10). TTA served as a negative control in this assay (Figure 4B, number 11). A semiquantitative RT-PCR experiment further showed that these mutations at codon position -23 did not affect the stability of the mRNAs derived from these constructs (Figure 4D).

### Initiation activities determined using *lacZ *as a reporter

**Figure 5:**
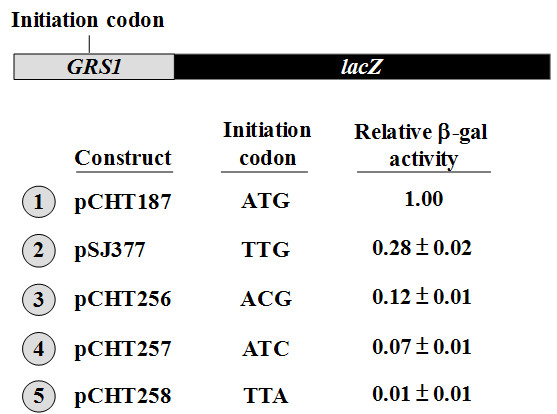
**Comparison of the efficiencies of various non-AUG initiator codons using *lacZ *as a reporter**. Efficiencies of translation using various initiator codons were determined by measuring the relative β-gal activities in extracts prepared from the transformants. The data were obtained from three independent experiments, and the relative β-gal activities are presented as the mean ± 2 × S.D., with the β-gal activity of the construct carrying an ATG initiator codon as a reference.

To verify whether the Western blot assays shown in Figure 4 faithfully reflect the initiation activities of the various non-AUG initiator codons, we next employed a different assay using *lacZ *as a reporter [21]. The *lexA *portion of the *GRS1-lexA *fusion constructs was replaced by an initiator mutant of *lacZ*, yielding various *GRS1-lacZ *fusion constructs (schematized in Figure 5). The β-gal activities derived from these fusion constructs were then determined. As shown in Figure 5, ATG, TTG, ACG, and ATC had relative initiation activities of 1.00: 0.28: 0.12: 0.07 (Figure 5, numbers 1~4), ratios which are very close to those determined by Western blotting (Figure 4). In contrast, no discernible β-gal activity was found for the TTA construct (Figure 5, number 5).

## Discussion

Despite significant differences in contextual preferences and sensitivities between non-AUG initiators of yeast and higher eukaryotes [21,27], our results show that except for AAG and AGG, all non-AUG codons that differ from AUG by a single nucleotide can act as initiator codons in yeast (Figure 2). An obvious advantage of beginning translation at non-AUG initiator codons is that these codons significantly vary in their initiation activity and are subject to regulation by the sequence context. As a consequence, they are more suitable than AUG to serve as alternative translation initiation sites to modulate the relative levels of two (or more) distinct protein isoforms [21]. While efficiencies of translation initiation from non-AUG codons are much lower (~10%~50%) than that from an AUG triplet positioned at the same site, the AlaRS or GlyRS protein initiated from these non-AUG codons was sufficient to rescue the growth defect of their respective knockout strains on YPG plates (Figs. 2, 4). Even though protein levels of the mitochondrial form of AlaRS can be drastically reduced, complementation functions at a fairly high efficiency. However, it should be noted that translation initiation from codons other than the often-seen non-AUG initiator codons does occur in nature. For example, a CUU triplet was reported to serve as a translation start site in an insect picorna-like virus via a mechanism known as an "internal ribosomal entry site" [28].

In higher eukaryotes, the sequence context can appreciably modulate the efficiency of translation initiation from AUG. In contrast, in low eukaryotes, the sequence context appears to have a negligible effect on translation initiation from AUG [29]. For example, Cigan *et al*., reported that sequence context changes at both 5' and 3' to the yeast *HIS4 *AUG initiator resulted in no more than a 2-fold decrease in expression [15]. However, recent studies argued that sequence context, in particular the nucleotide at position -3, plays a critical role in non-AUG initiation in yeast [21,24]. In this connection, it was interesting to point out that the non-AUG initiator codons of *ALA1 *and *GRS1 *and the cryptic initiator codon of *ALA1 *identified herein all bear a favorable nucleotide "A" at their relative position -3 [18,19]. On the other hand, having -3A alone does not guarantee that a non-AUG codon such as ATA can efficiently act as an initiator codon. Perhaps, the individual start codon mutations have different effects on stabilities of secondary structures around the start codon.

## Conclusion

Not all non-AUG codons that differ from AUG by a single nucleotide can act as initiator codons in yeast. In addition, a sequence context that is most favorable for a given non-AUG initiator codon might not be as favorable for another. Thus, it appears that every non-AUG initiator codon has its own favorite sequence context in yeast.

## Abbreviations

aaRS: aminoacyl-tRNA synthetase; *ADH*: alcohol dehydrogenase; AlaRS: alanyl-tRNA synthetase; FOA: 5-fluoroorotic acid; GlyRS: glycyl-tRNA synthetase; PCR: polymerase chain reaction; ValRS: valyl-tRNA synthetase; YPG: yeast extract-peptone-glycerol.

## Authors' contributions

CPC generated the various *ALA1 *constructs and performed the screening of functional non-AUG initiator codons, complementation assays, and RT-PCR assays. SJC generated the various *ALA1-lexA *fusion constructs and performed the Western blotting. CHL performed the β-galactosidase assays. TLW helped design the experiments. CCW coordinated the project and wrote the manuscript. All authors read and approved the final manuscript.
